# *pitpβ_w* Encoding Phosphatidylinositol Transfer Protein Is Involved in Female Differentiation of Chinese Tongue Sole, *Cynoglossus semilaevis*

**DOI:** 10.3389/fgene.2022.861763

**Published:** 2022-03-30

**Authors:** Yuxuan Sun, Mengqian Zhang, Peng Cheng, Zhihong Gong, Xihong Li, Na Wang, Min Wei, Xiaodong Xu, Wenteng Xu

**Affiliations:** ^1^ Laboratory for Marine Fisheries Science and Food Production Processes, Pilot National Laboratory for Marine Science and Technology, Yellow Sea Fisheries Research Institute, Chinese Academy of Fishery Sciences (CAFS), Qingdao, China; ^2^ Jiangsu Ocean University, Lianyungang, China; ^3^ Qingdao Vland Biotech Company Group, Qingdao, China

**Keywords:** *Cynoglossus semilaevis*, *pitpβ_w*, female differentiation, oogenesis, RNA interference

## Abstract

Phosphatidylinositol transfer protein (pitp) plays an important role in phospholipid transfer in animals. A pitp variant (*pitpβ_w*) in Chinese tongue sole was identified by transcriptomic analysis for its female-biased expression. The coding sequence of *pitpβ_w* was 816 bp, encoding a 371-amino-acid protein. *pitpβ_w* showed female-biased expression and was relatively high in brain, muscle, and ovary tissues. In different developmental stages of the ovary, *pitpβ_w* could be detected from 40 days until 3 years post hatching, and the highest expression was observed at 90 days. *In situ* hybridization revealed that *pitpβ_w* was predominantly localized in early-stage oocytes (I–III stages). After siRNA-mediated knockdown of *pitpβ_w* in an ovarian cell line, the expression of *sox9a* was reduced, while that of *figla_tv1* and *sox9b* was significantly increased. Our findings suggest that *pitpβ_w* might be involved in female differentiation and early oogenesis.

## Introduction

The gene-encoding phosphatidylinositol transfer protein (pitp) was discovered and named for its role in the transfer of phospholipids between membrane systems and the regulation of phospholipid signal transduction (Helmkamp et al., 1974). *pitp* is reported to function in various processes, such as neurodegeneration, intestinal absorption diseases, stress response, and developmental regulation (Wirtz, 1991; Cockcroft, 1999). As one of the three types of soluble pitp proteins in multicellular organisms (Hsuan and Cockcroft, 2001), the *Pitp* beta isoform (Pitpβ) is supposed to maintain the relative level between phosphatidylinositol (PI) and phosphatidylcholine (PC), while its functionality is poorly reported in comparison to the other two soluble members, *Pitpα* and *RdgB* (Cockcroft and Garner, 2013). In zebrafish, *pitpβ* is necessary for maintaining photoreceptor cell segments in the retina (Ile et al., 2010). In rats, *pitpβ* can be detected with high expression in the embryo and early postnatal stage, coincident with the formation of the nervous system and gray matter development (Utsunomiya et al., 1997). The content in the cells is approximately about 5–10 mmol/L accounting for 0.1% of the total protein in the brain cytoplasm (Hsuan and Cockcroft, 2001). These reports indicated the importance of *pitpβ* involved in neural development, but it remained unclear whether it was involved in other functions.

In this study, we have conducted a functional analysis of a *pitpβ_w* gene in Chinese tongue sole. As an economically important fish in Northeast Asia, Chinese tongue sole exhibits obvious sexual growth dimorphism where female individuals can grow 2–4 times faster than males; thus, understanding the sex differentiation mechanism would be pivotal for developing sex control technology and beneficial for the tongue sole industry (Chen et al., 2014). Many genes were reported to show female-biased expression, including *figla_tv1, r-spondin 1*, *lhx9*, and *zglp1*, and their possible role in ovarian development was investigated (Li et al., 2016; Li et al., 2017; Liu et al., 2018; Dong et al., 2019; Zhu et al., 2019). Recently, the study of female differentiation was greatly advanced by the progress of transcriptomic analysis. Lin et al. (2021) identified a number of genes involved in gonad differentiation in the tongue sole, such as the *dmrt1*, *amh*, and *sox* gene family. Combining lncRNA and mRNA transcriptome analyses, Dong et al. revealed several reproduction-related pathways and proposed that two lncRNAs were co-localized with *cyp17a1* and *cyp19a1* in the follicular cell layer (Dong et al., 2021). In our previous transcriptomic analysis, a set of genes was identified to show female-specific expression and to be involved in female differentiation (Xu et al., 2021). One of these genes, *pitpβ_w*, localized on the W chromosome, was chosen for further analysis including its expression pattern, cellular localization, and regulatory role in ovarian cell lines. The data would provide important information for understanding the molecular mechanism of sex differentiation in Chinese tongue sole.

## Materials and Methods

### Ethics Statement

The study was performed under the inspection of the committee at the Yellow Sea Fisheries Research Institute. MS-222 was used to anesthetize fish to minimize suffering during the experimental procedure.

### Fish Sampling

All fish used in these experiments were purchased from Huanghai Aquaculture Ltd. (Haiyang, China). To determine the genetic sex of the fish, small samples of the caudal fins were collected and used for genetic sex screening as previously described. In brief, genomic DNA was extracted from the fin and used as a template for PCR reaction. The resultant PCR products were examined by agarose gel electrophoresis. One band was observed for the male sample, while two bands were observed for the female samples (Liu et al., 2014). The muscle, spleen, liver, head kidney, intestine, heart, brain, and ovary were sampled from 1.5 years post hatching (yph) fish (three male and three female individuals). The average body size (BS) and weight (BW) were 21 cm, 45 g for male and 28 cm, 126 g for female. The tissues were immediately frozen with liquid nitrogen and then stored in a −80°C refrigerator until RNA extraction.

At the same time, gonadal samples at different developmental stages, which are 40, 60, and 90 days post hatching (dph), 6 months post hatching (mph), and 1.5 and 3 yph, were extracted and fixed in 4% paraformaldehyde at 4°C for 24 h and then stored in 70% ethanol at −20°C for *in situ* hybridization. Gonads at 40 and 60 dph were not differentiated, so samples from these two stages were obtained at the latter part of the visceral mass, which were the mixture of gonad, muscle, and other tissues. After genetic sex screening, only gonads from genetic female individuals were used for further analysis. Three individuals were used for analysis, and the average BS and BW for 40, 60, 90 dph, 6 mph, and 1.5 and 3 yph were 3 cm, 0.35 g; 4.5 cm, 0.69 g; 6 cm, 1.45 g; 15.7 cm, 22,4 g; 28 cm, 126 g; and 54 cm, 900 g, respectively.

### Sequence Analysis and Alignment

The coding sequence, molecular weight, and isoelectric point were calculated on http://web.expasy.org/. The protein domain was predicted by SMART (http://smart.embl-heidelberg.de/). A phylogenetic tree was constructed using MEGA 6.0 by employing the maximum likelihood method.

### cDNA Synthesis and Real-Time PCR (qPCR) Analysis

Total RNA was isolated using TRIzol reagent (Invitrogen, Carlsbad, CA) following the manufacturer’s instructions. The total RNA was reverse transcribed into cDNA using PrimeScript™ RT reagent kit (TaKaRa Bio Inc., Otsu, Japan), and gDNA Eraser was added to the resulting product to remove all DNA that might contaminate the genome. qPCR was used to determine the expression level of *pitpβ_w* in various tissues and at different developmental stages of the ovary. qPCR analysis was performed using a 7500 ABI real-time PCR instrument (Applied Biosystems, United States) with a TAKARA TB Green Premix Ex Taq II kit (TAKARA) following the manufacturer’s instructions. In brief, a 20 μL volume reaction system was prepared, containing 10.0 μL TB Green Premix Ex Tap, 0.4 μL ROX Dye II, 0.4 μL of each sense and anti-sense primer, 1.0 μL cDNA, and 7.8 μL ddH2O. The primers used in this study are shown in Table 1. The amplification procedure consisted of 30 s at 95°C and 40 cycles of 95°C for 5 s and 60°C for 34 s. Then, the default program of melting curve β-actin was used as an internal reference (Li et al., 2010). The transcriptional levels of genes were analyzed by the 2^△△Ct^ method (Livak and Schmittgen, 2001). Using the *t* test, significant differences were defined when *p* < 0.05.

**TABLE 1:** Primers used in the study.

Primer	Sequences (5′-3′)	Purpose	Product size
*pitpβ_w* F	AAAGAGCTGGTTAATAGC	qPCR	152 bp
*pitpβ_w* R	AAGAGTTGGCGATGAAAG		
sense F	TAA​TAC​GAC​TCA​CTA​TAG​GGG​ATT​TCT​ATA​AAA​GGC​AA	ISH	248 bp
sense R	GAAATAACATATGGAGAG		
antisense F	GATTTCTATAAAAGGCAA		
antisense R	TAA​TAC​GAC​TCA​CTA​TAG​GGG​AAA​TAA​CAT​ATG​GAG​AG		
*figla* F	ACA​TAG​AGA​AGT​TCA​AAC​GAG​CC	qPCR	210 bp
*figla* R	CGG​TAG​CAG​CTT​TTA​GTG​TGT​CT		
*sox9a* F	AAG​AAC​CAC​ACA​GAT​CAA​GAC​AGA	qPCR	150 bp
*sox9a* R	TAG​TCA​TAC​TGT​GCT​CTG​GTG​ATG		
*sox9b* F	AAG​AAC​CAC​ACA​GAT​CAA​GAC​AGA	qPCR	150 bp
*sox9b* R	TAG​TCA​TAC​TGT​GCT​CTG​GTG​ATG		
*cyp19a* F	GGT​GAG​GAT​GTG​ACC​CAG​TGT	qPCR	230 bp
*cyp19a* R	ACGGGCTGAAATCGCAAG		
*igf1* F	GTA​TCT​CCT​GTA​GCC​ACA​CCC​TCT	qPCR	137 bp
*igf1* R	GCC​TCT​CTC​TCC​ACA​CAC​AAA​CT		
*actin* F	CCT​TGG​TAT​GGA​GTC​CTG​TGG​C	qPCR	150 bp
*actin* R	TCC​TTC​TGC​ATC​CTG​TCG​GC		

### Localization of *pitpβ_w* mRNA in Gonadal Cells

*In situ* hybridization (ISH) was performed with the previously reported method to understand the expression pattern (Kobayashi et al., 2000). In brief, primers were designed for *pitpβ_w* to amplify 248 bp fragments (Table 1). The T7 binding sequence was included in the primer to facilitate the direct transcription by T7 RNA polymerase and generate digoxin (DIG) labeled probes. Sections were subjected to deparaffination and incubated with probes at 55°C overnight and then blocked for 4 h at room temperature. Anti-DIG-antibodies (Roche) were added for overnight incubation, and the signal was developed using nitrobluetetrazolium/5-bromo-4-chloro-3-indolyl phosphate (Roche, Mannheim, Germany).

### Small Interfering RNA-Mediated Interference in Ovarian Cell Lines

Specific *ptpβ-siRNA* targeting CAG​TTG​AGT​TTA​ATG​TTT​CAA​TG and non-specific siRNA used as a negative control (NC) were synthesized by Sangon Co., Ltd. (Shanghai, China). siRNA-mediated interference was performed according to the reported procedure in the tongue sole ovarian cell line (Sun et al., 2015; Li et al., 2016). In brief, the ovary from adult fish was collected and cut into small pieces (∼1 mm^3^), which were washed with PBS and then digested with trypsin solution (0.25% trypsin and 0.2% EDTA in PBS) for 10 min. After centrifugation at 180 *g* for 10 min, the cell pellet was suspended and seeded into a six-well plate at 24°C for 3 days when the cells formed a confluent monolayer. The cells were then subjected to the abovementioned 0.25% trypsin–EDTA solution and cultured for 3 days to allow the formation of a confluent monolayer again. Till date, the ovary cell line has been cultured for over 70 passages. The siRNA was transfected into ovarian cell lines using Lipofectamine 3,000 reagent following the manufacturer’s instructions. The effects at different time periods (24, 48, and 72 h) after interference were compared, and the 48 h sample showed the most significant silencing effect (data not shown). After interference for 48 h, qPCR was conducted to analyze the expression levels of *figla_tv1* (*KT966740.1*), *sox9a* (NM_001294243.1), *sox9b* (XM_008315177.3), *cyp19a* (NM_001294183.1), and *insulin-like growth factor* (*igf1*, NM_001294198.1) (primers listed in Table 1). All experiments were performed in triplicate.

## Results

### Cloning and Characteristics of *pitpβ_w*

**FIGURE 1:**
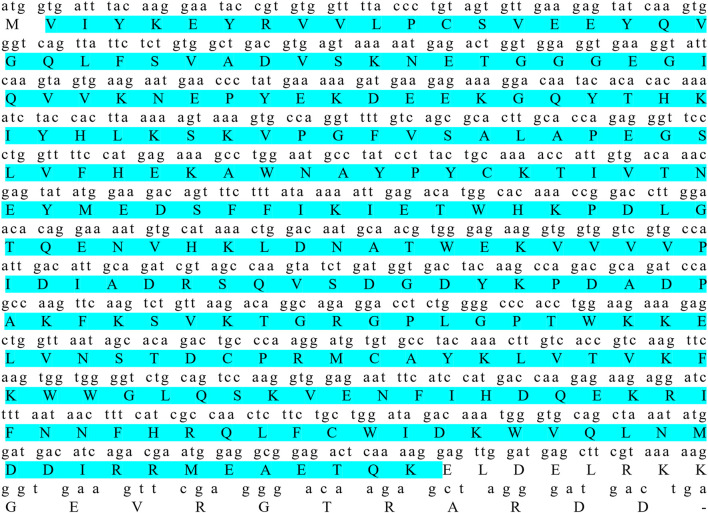
Coding sequence and deduced amino acid sequence of *pitpβ_w*. Amino acids in sky blue indicate the classic Pitp domain.

**FIGURE 2:**
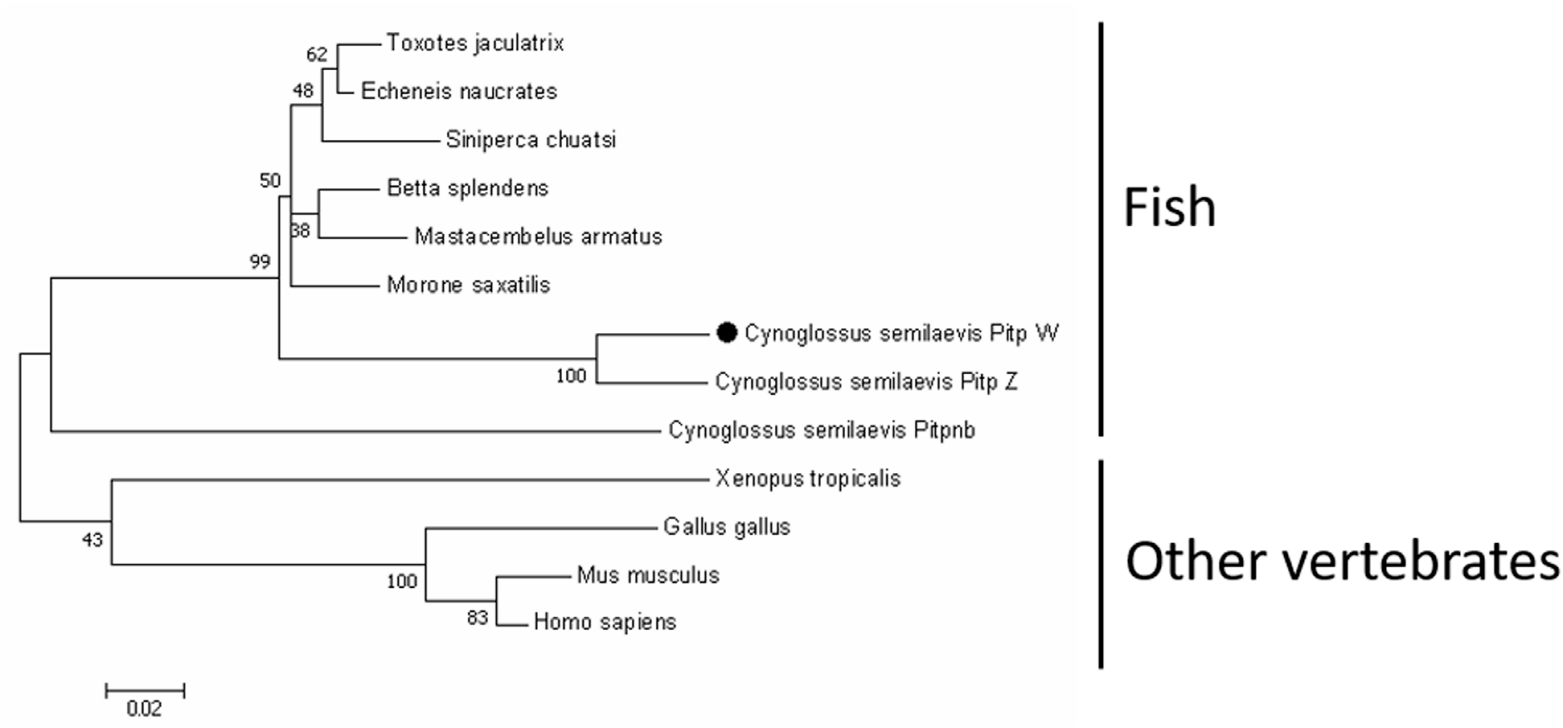
Phylogenetic analysis of *Pitpβ* from *C. semilaevis* and other vertebrates. Numbers at nodes represent NJ bootstrap values. The GenBank accession numbers were as follows: *Cynoglossus semilaevis* Pitp_w, XP_008333459.1; *Cynoglossus semilaevis* Pitp_z, XP_008334298.1; *Cynoglossus semilaevis* Pitpnb, XP_008323858.1; *Morone saxatilis*, XP_035514806.1; *Betta splendens*, XP_029019228.1; *Toxotes jaculatrix*, XP_040898022.1; *Siniperca chuatsi*, XP_044053196.1; *Echeneis naucrates*, XP_029366369.1; *Mastacembelus armatus*, XP_026177048.1; *Xenopus tropicalis*, NP_989122.1; *Gallus gallus*, NP_001383302.1; *Mus musculus*, AAA87593.1; and *Homo sapiens*, AAH18704.1.

As shown in Figure 1, the coding sequence of *pitpβ_w* was 816 bp (XM_008335237.3), encoding a 271-amino-acid (aa) protein with a predicted molecular weight of 31.52 kDa and an isoelectric point of 6.41. Based on SMART prediction, the protein composed a classic Pitp domain (aa 2-252). In the phylogenetic tree, vertebrate Pitpβ formed two clusters; fish Pitpβ including tongue sole Pitpβ_w formed one cluster, and another vertebrate Pitpβ formed the other cluster (Figure 2).

### Spatiotemporal Expression Pattern of *pitpβ_w*

**FIGURE 3:**
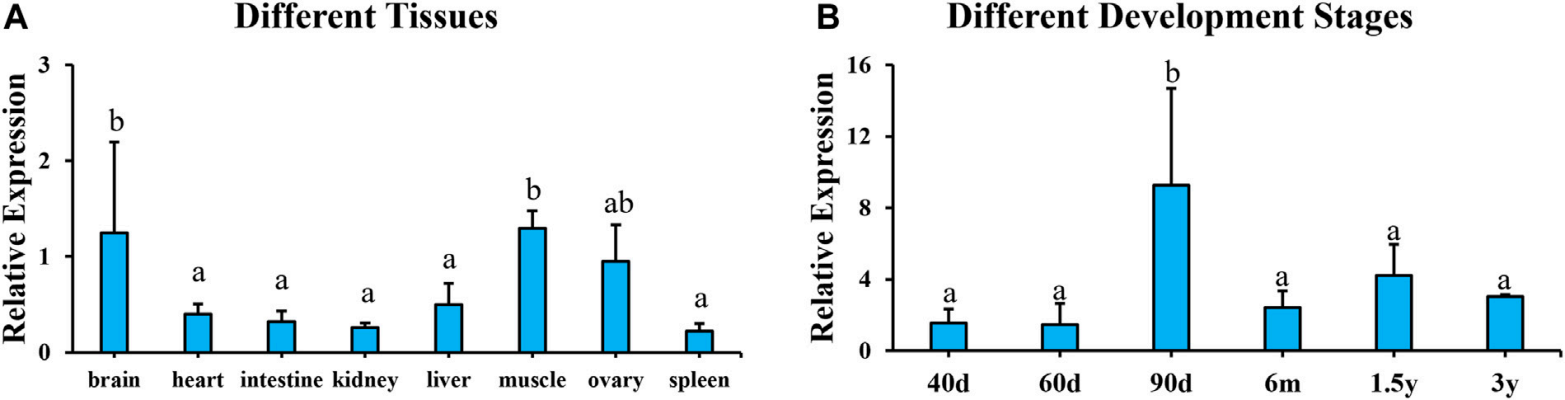
Relative expression of *pitpβ_w* in different tissues from female **(A)** and different developmental stages of the ovary **(B)**. Expression levels with different letters indicate a significant difference (*p* < 0.05).

To analyze the tissue distribution of *pitpβ_w*, qPCR was performed using eight different tissues from 1.5 yph tongue sole. No expression was detected in tissues from males (data not shown). In females, *pitpβ_w* was detected in all eight examined tissues, with the highest expression in the brain and muscle, followed by the ovary and other tissues (Figure 3A). The female-biased expression led us to analyze its profile at different developmental stages of the ovary. As shown in Figure 3B, *pitpβ_w* is expressed from 40 dph to 3 yph. The highest expression occurred at 90 dph.

### Cellular Localization of *pitpβ_w* in the Ovary

**FIGURE 4:**
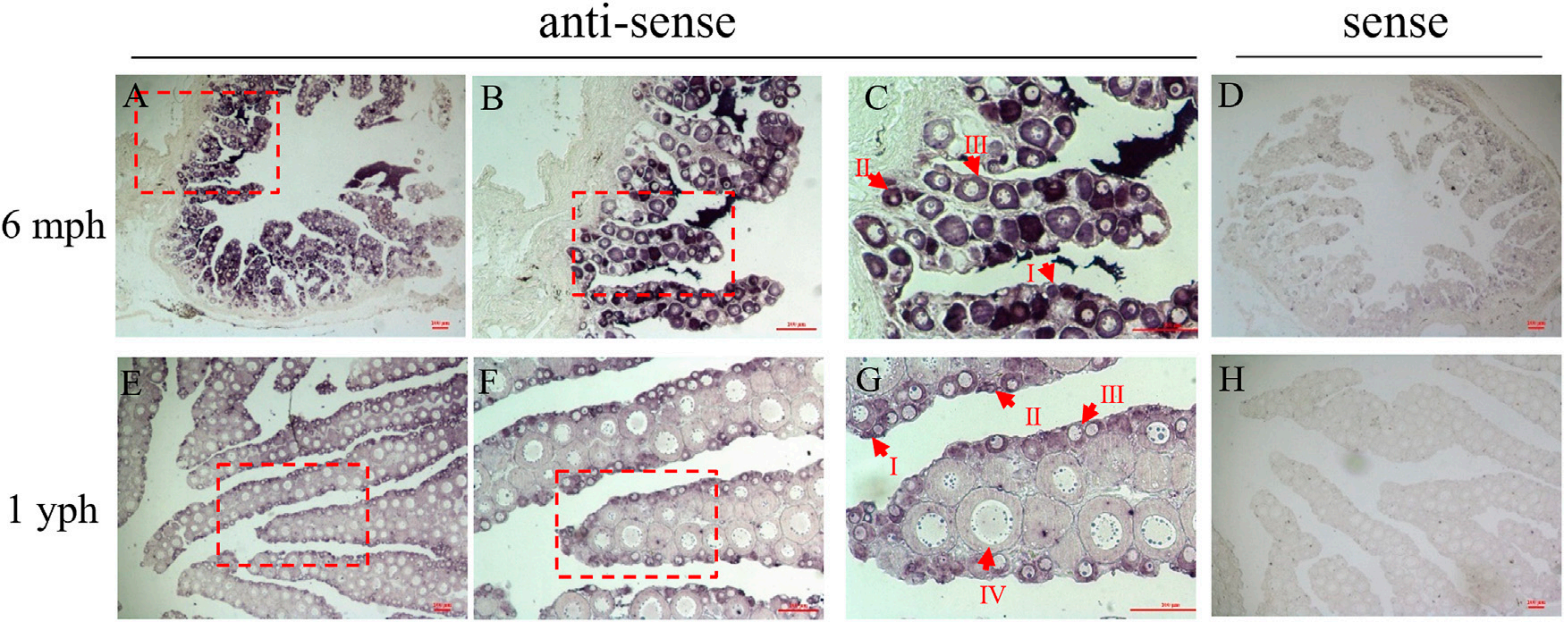
Cellular localization of *pitpβ_w* in the ovaries. **(A–C)** ISH in the 6 mph ovary with antisense probes corresponding to 40 ×, 100 ×, and 200 × magnification; enlarged areas are highlighted by a red frame; **(D)** ISH in the 6 mph ovary with sense probes; **(E–G)** ISH in the 1 yph ovary with antisense probes corresponding to 40ⅹ, 100ⅹ, and 200ⅹ magnification; enlarged areas are highlighted by a red frame; and (H) ISH in the 1 yph ovary with sense probes. Oocytes at different developmental stages are marked by I, II, III, and IV. Scale bars: 100 μm.

ISH was performed to investigate the cellular localization of *pitpβ_w* in the ovary. As shown in Figures 4A–C,E–G, the signals could be observed in the oocytes both at 6 mph and 1 yph. However, the signals seemed stronger in early-stage oocytes (I–III stages) than in stage IV (Figure 4G). Sense probes used as a negative control showed no significant signals (Figures 4D,H).

### *pitpβ_w* Knockdown and Its Effect on Sex-Related Genes

**FIGURE 5:**
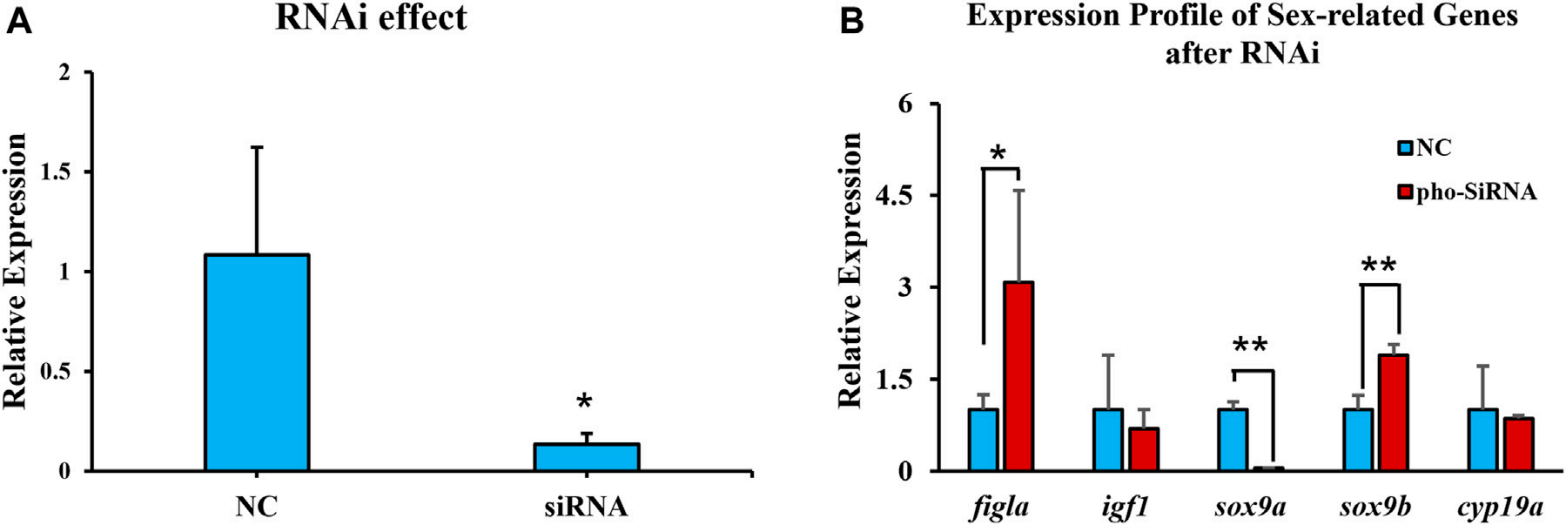
Expression levels of *pitpβ_w* and sex-related genes in the ovarian cell line after RNAi for 48 h **(A)** Expression of *pitpβ_w* after siRNA. **(B)** Expression of *figla_tv1*, *igf1*, *sox9a*, *sox9b*, and *cyp19a* after RNAi. NC, negative control group. siRNA, *rchy1-siRNA*-treated group. Asterisks indicate significant differences (*p < 0.05; **p < 0.01).

An ovarian cell line was employed to assess the *pitpβ_w* knockdown effect. After siRNA transfection for 48 h, *pitpβ_w* expression was reduced to approximately 13% (Figure 5A), suggesting an efficient knockdown effect. The sex-related genes were then evaluated, revealing that *sox9a* was reduced, while *figla_tv1* and *sox9b* were significantly increased (Figure 5B).

## Discussion

From the structural perspective, the pitp family can be divided into two classes. Class I contains single-domain proteins (*Pitpα*, *Pitpα*, and *RdgBβ*, all soluble), and class II consists of multiple-domain proteins (*RdgBαI* and *RdgBαII*). In Chinese tongue sole, there were five class I members, but no class II members were identified after genomic screening. This observation is similar to that in zebrafish, which has four class I members and one class II member (but lacks the pitp domain) (Elagin et al., 2000; Xie et al., 2005; Ile et al., 2010; Ashlin et al., 2018), suggesting that class I members in fish might be sufficient for phospholipid transfer. In Chinese tongue sole, the five class I members include one *pitpα*, one *rdgBβ*, and three *pitpβ* homologs, namely, *pitpβ_w* on chromosome W, *pitpβ_z* (XM_008336076.3) on chromosome Z, and *pitpnb* (XM_008325636.3) localized on chromosome 14. Although *pitpβ_w* transcription was specific to females and *pitpβ_z* and *pitpnb* transcription could be detected in both males and females (data not shown)*,* their transcripts and amino acids showed high similarity (Supplementary Figure S1). Especially for *pitpβ_z* and *pitpβ_w*, there were only 14 amino acid differences at the protein level (271 aa). In tongue sole, we have observed a set of genes with the expression patterns similar to those of *pitpβ_w* and *pitpβ_z*. These genes are localized on the Z and W chromosomes, respectively (Xu et al., 2021), producing similar transcripts and proteins but with distinguishable differences, and the transcripts from W chromosome-localized genes are usually female specific. Therefore, it would be interesting to test whether their functionality was redundant or divergent in the future.

Among the classic phosphatidylinositol transfer proteins, *pitpβ* function has been less studied than *pitpα* and *rdgBβ*, and its role in phospholipid transfer seemed dispensable. In this study, *pitpβ_w* showed female-biased expression, high expression at 90 dph (key stage for gonadal differentiation), and was mainly localized in early-stage oocytes (stages I, II, and III). In Chinese tongue sole, sex determination was proposed at approximately 50 dph, and gonadal differentiation was initiated at 56–62 dph. However, the cellular differentiation is lagging, and cellular differentiation usually occurs in the ovary at 90–120 dph, accompanied by the appearance of the ovarian cavity. Subsequently, the oocyte continues to differentiate and reaches the sexually mature stage at approximately 2 yph (Chen et al., 2014). Thus, high *pitpβ_w* expression at 90 dph was consistent with cellular differentiation in the ovary. Since there have been few reports regarding the function of *pitpβ* in sex differentiation, we conducted *in vitro* experiments to obtain some clues. Knockdown of *pitpβ_w* in an ovarian cell line resulted in the upregulation of *figla_tv1* and *sox9b* and downregulation of *sox9a*. As is known, *figla_tv1* in fish has been previously suggested to have a conserved function in oogenesis (folliculogenesis) (Kanamori et al., 2008; Yuan et al., 2014; Rouault et al., 2015; Li et al., 2016). In contrast, *sox9a* in fish is commonly accepted as a key modulator in male differentiation and testicular development (Berbejillo et al., 2012; Adolfi et al., 2015). Thus, we postulate that *pitpβ_w* might play an important role in female differentiation and early oogenesis by acting as a negative regulator. *sox9b* was recently identified as a *sox9a* homologue, and it showed high expression in the gonad of early-stage tongue sole compared to other tissues, so we proposed it was related to gonadal differentiation and, thus, examined its expression pattern after RNAi. *cyp19a* was not changed after RNAi which was unexpected. The unchanged expression might be due to the following reasons: 1) the rather short-term RNAi could not trigger the *cyp19a* response, or 2) *in vitro* experiment could not totally mimic the *in vivo* condition, so long-term RNAi or *in vivo* trials should be considered in the future. However, it showed unbiased expression between the testis and ovary, and the function of *sox9b* needs to be determined. Notably, the expression of *pitpβ_w* is high in brain and muscle tissues in addition to the ovary. It is possible that *pitpβ_w* could also function in phosphatidylinositol transfer and neural development (not the research focus in this study), and its regulatory role in ovarian differentiation may be coordinated by participating in the hypothalamic–pituitary–gonadal axis. In the future, we will perform RNAi using a brain cell line to provide a more complete picture. This approach will be ideal for conducting *in vivo* trials, such as gene editing, to address the remaining unresolved issues (Dong et al., 2021).

## Data Availability

The original contributions presented in the study are included in the article/Supplementary Material, further inquiries can be directed to the corresponding author.

## Supplementary Material

Supplementary Figure S1 Alignment of the three Pitpβ in Chinese tongue sole. Depending on different extents of conservation, the sites are labelled in black, grey, and white.
