# Supplementary figures and images for: pitpβ_w Encoding Phosphatidylinositol Transfer Protein Is Involved in Female Differentiation of Chinese Tongue Sole, Cynoglossus semilaevis
Source: Front Genet. 2022 Mar 30;13:861763. doi: 10.3389/fgene.2022.861763 (PMC9006047; doi:10.3389/fgene.2022.861763)

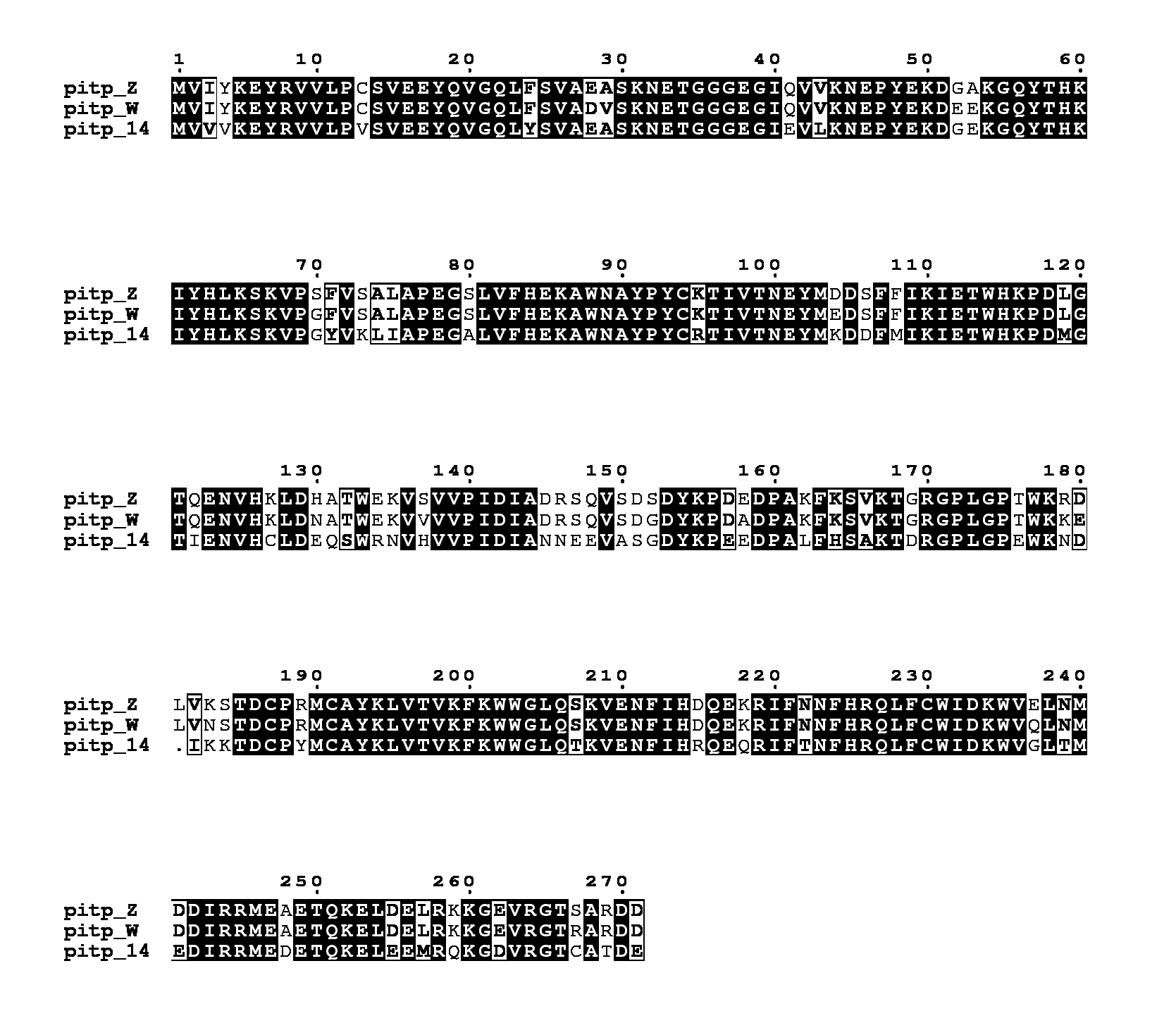

Supplement: Supplementary file 1 [file Image1.tiff]
